# The High Prevalence of Diabetes in a Large Cohort of Patients Drawn From Safety Net Clinics

**DOI:** 10.5888/pcd13.160056

**Published:** 2016-06-16

**Authors:** Gregory A. Nichols, MaryAnn McBurnie, Ludmilla Paul, Jennifer E. Potter, Sheila McCann, Kenneth Mayer, Gerardo Melgar, Sele D’Amato, Jennifer E. DeVoe

**Affiliations:** Author Affiliations: MaryAnn McBurnie, Kaiser Permanente Center for Health Research, Portland, Oregon; Ludmilla Paul, Sheila McCann, Health Choice Network of Florida, Miami, Florida; Jennifer E. Potter, Beth Israel Deaconess Medical Center, Boston, Massachusetts; Kenneth Mayer, Beth Israel Deaconess Medical Center, and The Fenway Institute, Boston, Massachusetts; Gerardo Melgar, Cowlitz County Family Health Center, Longview, Washington; Sele D’Amato, OCHIN Patient Engagement Panel, Portland, Oregon; Jennifer E. DeVoe, Oregon Health and Science University, and OCHIN, Portland, Oregon.

## Abstract

**Introduction:**

Underserved populations have been overlooked or underrepresented in research based on data from diabetes registries. We estimated diabetes prevalence using a cohort developed from the electronic health records of 3 networks of safety net clinics that provide care to underserved populations.

**Methods:**

ADVANCE (Accelerating Data Value Across a National Community Health Center Network) is a partnership of the OCHIN Community Health Information Network (OCHIN), the Health Choice Network (HCN), and the Fenway Health Institute (FHI), representing 97 federally qualified health centers (FQHCs) and 744 clinic sites in 22 US states. Among 952,316 adults with a body mass index (BMI) measurement and at least 2 outpatient visits in 2012 to 2014, we calculated diabetes prevalence using outpatient diagnoses, diagnostic laboratory results, or dispenses of anti-hyperglycemic agents no more than 730 days apart. We calculated prevalence by age, sex, race, Hispanic ethnicity, and BMI class.

**Results:**

The crude prevalence of diabetes was 14.4%. Men had a higher prevalence than women (16.5% vs 13.2%); diabetes prevalence increased across age categories. White patients had the lowest prevalence (11.4%) and Hawaiian/Pacific Islanders, the highest prevalence (21.9%), with prevalence ranging from 15.2% to 16.5% for other race/ethnicities. The association between BMI class and diabetes prevalence was similar across all racial/ethnic groups.

**Conclusion:**

The ADVANCE diabetes cohort offers an opportunity to conduct epidemiologic and comparative effectiveness research on underserved and underrepresented individuals, who have a higher prevalence of diabetes than the general US population.

## Introduction

Diabetes is a chronic condition for which multiple office visits, laboratory tests, and pharmacotherapies are recommended on a more than annual basis. As such, the care of patients with diabetes generates a great deal of data that, when available electronically, allows for the creation of registries and the evaluation of trends in diabetes prevalence and clinical management. Indeed, many examples exist, including longstanding health system–specific registries in both the private and public sectors in the United States and national registries in Europe (1–8). Although these and other diabetes registries were originally derived from administrative data, the widespread adoption of electronic health records (EHRs) has allowed for improved accuracy of diabetes identification as well as the creation of networked registries that span multiple health systems (9–11). Recent research has demonstrated the value of identification methods that include comprehensive EHR data (12).

To our knowledge, comprehensive diabetes registries to date have been created only of insured populations. A diabetes registry of patients at safety net clinics would comprise individuals who are underinsured or uninsured or otherwise vulnerable because of economic status, sexual orientation or gender-identity minority status, low literacy, functional or developmental limitations, or other barriers to accessing health care. These subgroups are typically omitted from or underrepresented in registries created only from medical records of insured individuals, because EHR data from safety net clinics have, until recently, been unavailable. Furthermore, most of the aforementioned subgroups are known to be at elevated risk of developing diabetes. Our objective was to create and describe a diabetes registry from the EHRs of 3 networks of safety net clinics and to calculate and compare diabetes prevalence by age, sex, racial/ethnicity, and body mass index (BMI) class. The result is a valuable resource for studying diabetes in these populations as well as filling critical gaps in knowledge that result from the absence of underinsured and uninsured individuals in registry-based studies.

## Methods

ADVANCE (Accelerating Data Value Across a National Community Health Center Network) is a partnership of the Community Health Information Network (OCHIN), the Health Choice Network (HCN), and the Fenway Health Institute (FHI). Specifics of ADVANCE and its components have been described elsewhere (13). Briefly, these 3 organizations together represent 97 federally qualified health centers (FQHCs) and 744 clinics in 22 US states, currently providing care to over 1.6 million people. ADVANCE was one of the first 11 clinical data research networks (CDRNs) funded by the Patient-Centered Outcomes Research Institute. Each CDRN was required to create a “weight cohort” of patients within their networks and a “common disease cohort” of their own choosing; ADVANCE chose diabetes as its common disease. The Western Institutional Review Board reviewed and approved the study.

To be included in the ADVANCE weight cohort, adult patients aged 20 or older were required to have at least one outpatient visit to an OCHIN, HCN, or FHI clinic in 2012, 2013, or 2014 with a body weight measured in that 3-year period and a height measured at any time in 2014 or earlier (n = 1,101,640). We used the earliest available weight in the period to calculate body mass index (BMI). We created the ADVANCE diabetes cohort as a subset of the weight cohort using the method described by SUPREME-DM (SUrveillance, PREvention and ManagEment of Diabetes Mellitus) study, except that we were unable to include inpatient diagnoses as an identification criterion (14). This method requires the presence of any combination of 2 “events” from outpatient diagnoses (ICD-9 code 250.x), diagnostic laboratory results (HbA1c >6.4%, fasting glucose >125 mg/dL, random glucose >199 mg/dL), or dispensation of anti-hyperglycemic agents (primarily metformin, sulfonylureas, or insulin) no more than 730 days apart. This method does not differentiate between type 1 and type 2 diabetes but captures patients with either type. To be considered for inclusion in the diabetes cohort, we restricted the weight cohort to people with at least 2 outpatient visits in the study period (n = 952,316). In addition to BMI, we extracted age, sex, race, and Hispanic ethnicity from the enrollment records of the participating health systems.

We calculated prevalence of diabetes by using adults with qualifying visits and BMI measurements as the denominator and people meeting diabetes criteria as the numerator. Because data on race/ethnicity are missing for 2.5% of the sample, we estimated prevalence for racial categories and Hispanic ethnicity only for those for whom these data were available. *P* values for all comparisons here were statistically significant because of our large sample size.

## Results

Of the 952,316 patients in the ADVANCE weight cohort, 559,134 (58.7%) were from OCHIN clinics, 373,555 (39.2%) were from HCN clinics, and 19,627 (2.1%) were from FHI (Table 1). Age and sex were similar for OCHIN and HCN patients. Because FHI provides care to the LGBT community (in addition to the broader population), these patients were much younger than patients from the other clinics and more likely to be men. About 58% of OCHIN patients were white, compared with 30% of HCN’s and 72% of FHI’s. BMI was similar for OCHIN and HCN and noticeably lower for FHI.

**Table 1:** Characteristics of the ADVANCE Population Used to Identify Diabetes, by Participating Health System, 2012–2014

Characteristics	FHI	HCN	OCHIN	Total
Total patients, n	19,627	373,555	559,134	952,316
Mean age, y (SD)	39.1 (13.5)	45.7 (15.4)	46.8 (16.5)	46.2 (16.1)
**Age group, y**				
20–44	67.2%	48.4%	48.0%	48.5%
45–64	28.8%	41.6%	37.7%	39.0%
≥65	4.1%	10.1%	14.3%	12.5%
**Sex**				
Men	62.9%	34.1%	39.3%	37.7%
Women	37.1%	65.9%	60.7%	62.3%
**Race/ethnicity**				
American Indian/Alaska Native	0.2%	0.2%	0.6%	0.4%
Asian	6.0%	1.8%	3.0%	2.6%
Non-Hispanic black	7.3%	22.2%	12.9%	16.5%
Hispanic or Latino	9.5%	43.1%	22.1%	30.1%
Multiple race	1.8%	0.7%	0.5%	0.6%
Native Hawaiian/Other Pacific Islander	0.5%	0.4%	0.3%	0.4%
Unknown	1.5%	2.0%	2.9%	2.5%
Non-Hispanic white	71.9%	29.6%	57.7%	47.0%
Other	1.4%	0.0%	0.1%	0.1%
Mean body mass index, kg/m^2^ (SD)	26.8 (5.6)	29.9 (7.3)	29.6 (7.4)	29.7 (7.4)
**BMI class**				
Underweight (<18.5 kg/m^2^)	3.4%	3.8%	3.4%	3.6%
Normal weight (18.5–24.9 kg/m^2^)	41.0%	23.5%	26.0%	25.3%
Overweight (25–29.9 kg/m^2^)	33.5%	31.3%	30.7%	31.0%
Class I obesity (30–34.9 kg/m^2^)	13.7%	21.8%	20.6%	20.9%
Class II obesity (35–39.9 kg/m^2^)	5.1%	10.7%	10.4%	10.4%
Class III obesity (≥40 kg/m^2^)	3.3%	8.8%	8.8%	8.7%

Abbreviations: ADVANCE, Accelerating Data Value Across a National Community Health Center Network; OCHIN, Community Health Information Network; HCN, Health Choice Network; FHI, Fenway Health Institute; SD, standard deviation; kg/m^2^, kilograms divided by height in meters squared.

**Figure 1:**
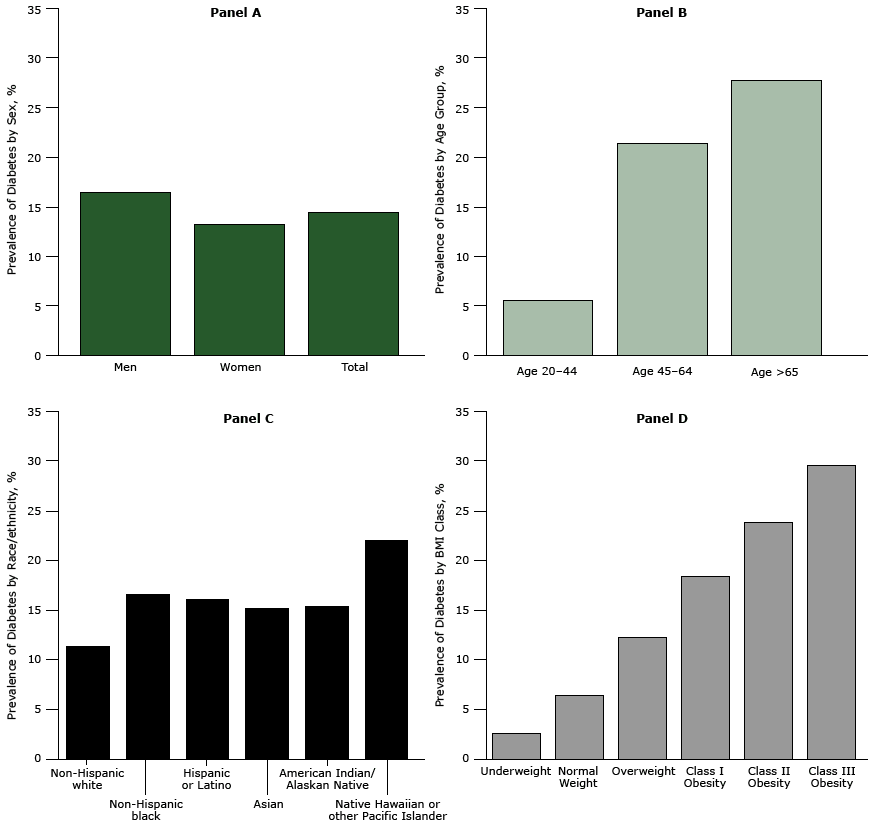
Prevalence of diabetes among ADVANCE study patients by sex and in total (Panel A); age group (Panel B); race/ethnicity (Panel C); and BMI class (Panel D). Body mass index was calculated as measured weight in kilograms divided by height in meters squared. **Table:** 

**Figure 2:**
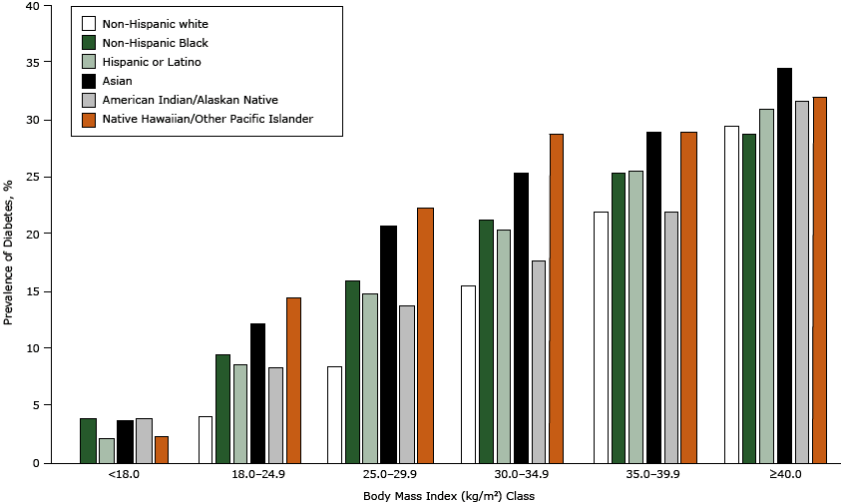
Prevalence of diabetes by body mass index class for selected races and Hispanic ethnicity. Body mass index was calculated as measured weight in kilograms divided by height in meters squared. **Table:** 

There were 137,445 patients with diabetes in the ADVANCE weight cohort (14.4%), (Table 2). Patients with diabetes were considerably older and more likely to be male and nonwhite than were patients without diabetes. About 62% of patients with diabetes were obese compared with 36.5% of patients without diabetes (*P* <.001). The 4 panels in Figure 1 show the crude prevalence of diabetes for people in various demographic strata. Men had a higher prevalence than women (16.5% vs 13.2%, Panel A), and diabetes prevalence increased across age categories (Panel B) (*P* <.001 for all comparisons). Non-Hispanic white patients had the lowest prevalence (11.4%) and Native Hawaiian or Other Pacific Islanders had the highest prevalence (21.9%). Diabetes prevalence of patients from other races ranged from 15.2% to 16.5% (Panel C) (*P* <.001 for all comparisons). The relationship between BMI class and diabetes prevalence was consistently linear (Panel D) and similar for all racial/ethnic groups (Figure 2).

**Table 2:** ADVANCE Patients by Diabetes Status, 2012–2014

Patient Characteristics	No Diabetes	Diabetes
Total patients, N	814,871	137,445
Percentage of total	85.6%	14.4%
**Age group, y**		
20–44	53.6%	18.3%
45–64	35.9%	57.8%
≥65	10.5%	23.9%
**Sex**		
Men	36.8%	43.2%
Women	63.2%	56.8%
**Race/ethnicity**		
American Indian/Alaska Native	0.4%	0.5%
Asian	2.5%	3.0%
Non-Hispanic black	15.7%	20.7%
Hispanic or Latino	29.2%	35.1%
Multiple race	0.6%	0.5%
Native Hawaiian/other Pacific Islander	0.3%	0.6%
Unknown	2.7%	1.7%
Non-Hispanic white	48.5%	37.9%
Other	0.1%	0.0%
**BMI class, kg/m^2^**		
Underweight (<18.5)	4.1%	0.7%
Normal weight (18.5–24.9)	27.7%	11.3%
Overweight (25–≥29.9)	31.8%	26.4%
Class I obesity (30–34.9)	20.0%	26.7%
Class II obesity (35–39.9)	9.3%	17.2%
Class III obesity (≥40)	7.2%	17.8%

Abbreviation: ADVANCE, Accelerating Data Value Across a National Community Health Center Network.

Diabetes Prevalence	
**Panel A. By Sex**	
Men	16.5%
Women	13.2%
Total	14.4%
**Panel B. By Age Group, y**	
Aged 20-44	5.5%
Aged 45-64	21.4%
Aged ≥65	27.7%
**Panel C. By Race/Ethnicity**	
Non-Hispanic white	11.4%
Non-Hispanic black	16.5%
Hispanic or Latino	16.1%
Asian	15.2%
American Indian/Alaska Native	15.3%
Native Hawaiian/Other Pacific Islander	21.9%
**Panel D. BMI Class**	
Underweight (<18.5 kg/m^2^)	2.6%
Normal weight (18.5–24.9 kg/m^2^)	6.4%
Overweight (25–29.9 kg/m^2^)	12.3%
Class I obesity (30–34.9 kg/m^2^)	18.4%
Class II obesity (35–39.9 kg/m^2^)	23.8%
Class III obesity (≥40 kg/m^2^)	29.6%

Race/ethnicity	Body Mass Index (kg/m^2^) Class					
	<18	18–24.9	25–29.9	30–34.9	35–39.9	≥40
Non-Hispanic white	2.4%	4.2%	8.6%	15.5%	22.0%	29.6%
Non-Hispanic Black	4.0%	9.6%	16.1%	21.3%	25.5%	28.8%
Hispanic or Latino	2.2%	8.7%	14.9%	20.5%	25.6%	31.0%
Asian	3.8%	12.3%	20.9%	25.4%	29.0%	34.7%
American Indian/Alaskan Native	4.0%	8.4%	13.9%	17.8%	22.0%	31.8%
Native Hawaiian/Other Pacific Islander	2.4%	14.5%	22.3%	28.9%	29.0%	32.1%

## Discussion

The ADVANCE CDRN assembled a cohort of 137,445 adults with diabetes who receive medical care from safety net clinics located in 22 states (6 western states, 2 southwestern states, 2 Midwest states, 4 states in the Great Lakes region, 4 southern states, 2 mid-Atlantic states, Alaska and Hawaii), providing a unique nationwide diabetes registry for conducting patient-centered outcome research among populations that are generally underrepresented in clinical trials and registry-based studies (11,13). In particular, the ADVANCE diabetes cohort allows for the study of diabetes and its complications among uninsured and vulnerable individuals, about whom relatively little is known.

Overall, the crude prevalence of diabetes in the ADVANCE cohort was 14.4%, somewhat higher than the 12.3% reported in the general US adult population (15). This finding can be partially explained by the larger proportion of high-risk subgroups in ADVANCE. For example, although the ADVANCE diabetes prevalence rates were similar to US rates for Hispanics (16.1% vs 17.0%) and non-Hispanic blacks (16.5% vs 14.7%), the proportions of the ADVANCE population that are Hispanic (30.1%) or non-Hispanic black (16.5%) are higher than their proportions in the US population. Although US diabetes prevalence may have increased since 2005 to 2006, crude and age-adjusted rates of diagnosed diabetes have leveled off since 2008 (16). Diabetes prevalence among Asians was only slightly lower than among blacks or Hispanics. Though counterintuitive, this finding is consistent with a report of a similar finding when Asians are aggregated but also demonstrated wide variation of diabetes prevalence among Asians when disaggregated into Chinese, Japanese, Filipino, and South Asian subgroups (17). We cannot provide that level of detail.

Diabetes prevalence and obesity are inextricably entwined, with clear evidence that obesity and weight gain increases the risk of developing diabetes (18,19). Higher diabetes prevalence in the ADVANCE cohort is likely due in part to the cohort’s having obesity rates that are substantially higher (40.0% vs 32.2%) than in the general US adult population (20). In our data, the relationship between obesity class and diabetes prevalence was remarkably linear and consistent across all racial/ethnic groups. Thus, despite the known increased risk of diabetes associated with minority groups, the impact of obesity on diabetes risk appears to be uniform. In addition to the health and economic burden of obesity, the risk of mortality increases among the obese despite improving trends in cardiovascular risk factor control (21,22). Recent analyses suggest that the previously increasing trends in obesity are leveling off in the general US population (20,23); it is unknown whether a similar plateau is also occurring in safety net populations. Nevertheless, despite a flattening of obesity trends, there are now more overweight and obese young people than ever before (20). Because earlier onset of obesity increases the risk of diabetes, we may witness a second wave of the diabetes epidemic in the near future (24).

Insurance status may also play a role in our study’s higher prevalence rates of diabetes. Although directly comparable data are elusive, about 16% of the US adult population did not have health insurance in 2012, whereas approximately 25% of the ADVANCE cohort was uninsured (25). Uninsured diabetes patients are less likely to receive preventive care or to know about their diabetes, and more likely to have poor glycemic control (26–28). Studies of the long-term ramifications of these disparities are needed and require datasets such as the ADVANCE data warehouse to execute them properly. The Affordable Care Act will provide insurance to many of those currently lacking it. Early indications are that uninsured community health center visits have substantially decreased and that more Medicaid patients with diabetes are receiving a diagnosis and being treated earlier (25,29). The long-term implications of such changes demand careful evaluation. We could not determine insurance status on an individual level, primarily because it can change from one encounter to another.

In addition to the uniqueness of the ADVANCE cohort, strengths include its size and the use of methods to identify diabetes that are consistent with the SUPREME-DM cohort and other electronic database definitions that capture both patients with diagnosed diabetes and patients with undiagnosed diabetes (12,29). Consistency with other cohorts presents the opportunity for collaborative efforts that could create a comprehensive and nationally representative database of all patients with diabetes.

There are also limitations to consider. First, inpatient data are a work-in-progress in ADVANCE and are not available. Although this limits our ability to examine some outcomes, lack of inpatient diagnoses has little impact on the identification of patients with diabetes (12). Second, similar to all diabetes registries and cohorts derived from EHR or administrative data, the ADVANCE cohort comprises clinic attendees who sought health care, thus excluding patients with undiagnosed and untreated disease. Because patients who do not routinely access health services are more likely to be from racial/ethnic minority communities, the diabetes estimates in this safety net CHC sample may underrepresent the prevalence of the disease in the most disenfranchised populations. Conversely, because the ADVANCE cohort is created from people with clinic contacts, it differs from health system-based cohorts such as SUPREME-DM that can include total enrollment as the denominator. This difference may have artificially increased prevalence estimates in ADVANCE. Furthermore, by requiring patients to have a BMI on record, we may have overselected patients who have diabetes or are at risk for its development. There may also be variation among sites represented in the 3 ADVANCE networks regarding data capture and clinician coding and documentation of diabetes. However, the SUPREME-DM algorithm that we used is a robust method that identifies more patients with diabetes than other electronic phenotype definitions (12). This is due in part to the inclusion of laboratory data that captures patients who may not have received a diagnosis (30). Unfortunately, the algorithm does not differentiate between type 1 and type 2 diabetes, and differences by diabetes type may exist. Finally, we believe that insurance status may have played a role in our results but cannot reliably differentiate between uninsured, underinsured, and fully insured patients on an individual level.

Despite these limitations, the ADVANCE diabetes cohort offers an opportunity to conduct epidemiologic and comparative effectiveness research in underserved and underrepresented individuals. This resource provides the ability to quickly identify safety net patients for randomized studies, to examine longitudinal trends, and to assess care practices and identify potential disparities within and across registries. As part of the larger PCORnet (National Patient-Centered Clinical Research Network) that is using identical methods across other CDRNs, and by using methods similar to SUPREME-DM, enormous opportunity exists for collaborations that could generate a more representative national database of people with diabetes, thus providing results that are more generalizable to the US population.
