# Erratum, Vol. 13, June 16 Release

**DOI:** 10.5888/pcd13.160056e

**Published:** 2016-09-15

In the article “The High Prevalence of Diabetes in a Large Cohort of Patients Drawn From Safety Net Clinics,” some figure notations and table footnotes were incorrect. Corrections were made to our website on June 21, 2016, and appear online at http://www.cdc.gov/pcd/issues/2016/16_0056.htm. We regret any confusion or inconvenience these errors may have caused.

